# Niche Modeling May Explain the Historical Population Failure of *Phytoseiulus persimilis* in Taiwan: Implications of Biocontrol Strategies

**DOI:** 10.3390/insects12050418

**Published:** 2021-05-06

**Authors:** Jhih-Rong Liao, Chyi-Chen Ho, Ming-Chih Chiu, Chiung-Cheng Ko

**Affiliations:** 1 Department of Entomology, National Taiwan University, Taipei 106332, Taiwan; d04632007@ntu.edu.tw (J.-R.L.); kocc2501@ntu.edu.tw (C.-C.K.); 2 Taiwan Acari Research Laboratory, Taichung 413006, Taiwan; mtho2005@yahoo.com.tw; 3 Center for Marine Environmental Studies (CMES), Ehime University, Matsuyama 7908577, Japan

**Keywords:** predatory mites, ecological niche, establishment

## Abstract

**Simple Summary:**

*Phytoseiulus persimilis* Athias-Henriot, a mite species widely used in pest management for the control of spider mites, has been commercialized and introduced to numerous countries. In the 1990s, *P. persimilis* was imported to Taiwan, and a million individuals were released into the field. However, none have been observed since then. In this study, we explored the ecological niche of this species to determine reasons underlying its establishment failure. The results indicate that *P. persimilis* was released in areas poorly suited to their survival. To the best of our knowledge, the present study is the first to predict the potential distribution of phytoseiids as exotic natural enemies. This process should precede the commercialization of exotic natural enemies and their introduction into any country.

**Abstract:**

Biological control commonly involves the commercialization and introduction of natural enemies. *Phytoseiulus persimilis* Athias-Henriot, a mite species widely used in the control of spider mites, was imported to Taiwan in the 1990s and was mass-reared and released into the field. However, none have been observed in comprehensive surveys of phytoseiid mites for over 30 years. In this study, the distribution of *P. persimilis* in Taiwan was predicted, and environmental variables that affect its distribution were analyzed. The mountainous region of southcentral Taiwan was determined to be suitable for the establishment of this species, whereas the four sites at which it was released in the 1990s, particularly those in southwestern Taiwan, exhibited low suitability. Notably, the minimum temperature of the coldest month was identified as a crucial limiting factor affecting the distribution of *P. persimilis*, indicating that a Mediterranean climate is more suitable for this species. To the best of our knowledge, this study is the first to predict the suitable distribution of exotic predatory mites in a biological control program. The present findings serve as a pivotal assessment framework for the commercialization and foreign introduction of natural enemies.

## 1. Introduction

For more than a century, numerous species of natural enemies have been commercialized, mass-reared, introduced, and released into the field for pest management and control, with successful results in both classical and augmentative biological control programs [1,2,3,4]. For example, the host-specific parasitoid *Anagyrus lopezi* De Santis was released to control the mealybug *Phenacoccus manihoti* Matile-Ferrero and is now established in tropical Thailand [5]. In addition, *Eurytoma erythrinae* Gates and Delvare was released to control *Quadrastichus erythrinae* and has been established in the tropical islands of Hawaii [6]. However, the establishment and persistence of these pest control species remains a serious problem in biological control programs [7]. Moreover, exotic biological control agents may adversely affect native or naturalized populations and in turn threaten endemic species through intraguild predation, as in the case of *Harmonia axyridis* (Pallas), commonly named the harlequin ladybird and also known as “the most invasive ladybird on Earth” [8]. This ladybird has been introduced worldwide as a classical biological control agent for hemipteran pests. However, the introduction of this species became catastrophic because they competed for prey with native natural enemies, became themselves a pest on fruit plants, and created a nuisance when they inhabited human dwelling in large numbers [8]. Therefore, the assessment of risks posed by exotic natural enemies (e.g., to the environment and non-target organisms) and the possibility of population establishment warrants urgent attention [1,2,9].

Ecological niche modeling, also known as species distribution modeling, uses computer algorithms, mathematics, and statistics to establish prediction models based on ecological niche theory [10,11]. Ecological niche modeling explores species niches or environmental spaces based on known environmental characteristics and species distribution data [10,12]. The maximum entropy method (MaxEnt), a machine learning approach, makes predictions of species distributions by analyzing species–environment relationships through the use of presence-only data and environmental variables [10]. As only presence data are required, this method has multiple applications, including the determination of suitable habitats for conservative species [10], predicting the distribution of invasive species [13], and modeling distribution shifts caused by climatic changes [14]. In these studies, the distribution of invasive pests in non-invaded areas was predicted using global presence-only data [15,16]. Slatculescu et al. [17] predicted the distribution of ticks (vectors of Lyme disease) using MaxEnt and constructed maps of environmental risk in southern and eastern Ontario, Canada. Negrini et al. [18] generated a risk map of *Steneotarsonemus spinki* Smiley in rice-growing areas around the globe using MaxEnt. However, to the best of our knowledge, there are as yet no published studies on phytoseiid mites based on MaxEnt.

**Figure 1:**
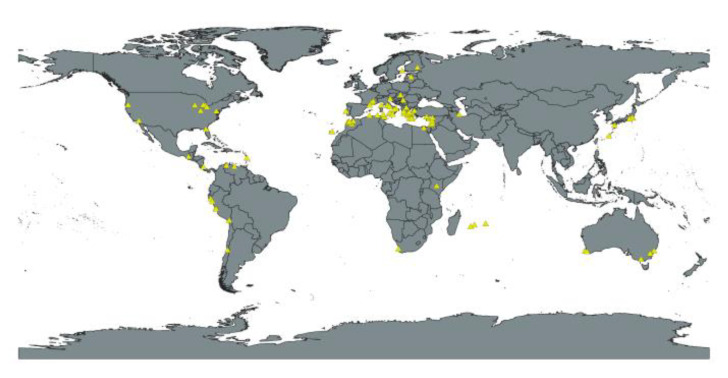
Global occurrence of *Phytoseiulus persimilis*. The triangles represent the distributions.

Certain species belonging to the mite family Phytoseiidae (Acari: Mesostigmata) have been used as biological control agents against mite pests as well as against small insects such as aphids, thrips, and whiteflies [19,20,21]. Predatory mites have been introduced in numerous countries. Twelve phytoseiid mite species are included on the “positive list” of “safe use biological control” agents of the European and Mediterranean Plant Protection Organization; for example, *Phytoseiulus persimilis* Athias-Henriot and *Neoseiulus californicus* (McGregor)] [22]. They can be introduced and used safely without harm to the environment; however, the absence of a species from the list does not imply that a species is unsafe but may indicate a lack of sufficient research on this species. *P. persimilis*, a specialist predator of *Tetranychus* mites, has been extensively used for commercial pest control, especially against *Tetranychus urticae* Koch, a major pest of numerous crop species in temperate and tropical countries [23,24]. This species originates from Algeria [23] and is mainly distributed in the Mediterranean basin (Figure 1, Table S1) [24,25,26], where the climate is characterized by mild rainy winters and warm to hot dry summers [27]. Previous studies have revealed that temperature and precipitation directly affect the growth and distribution of *P. persimilis*, which thrives in warm and highly humid environments [27,28,29]. 

*P. persimilis* has been introduced and established in numerous countries such as Japan [30], Egypt [31], and New Zealand [25] for biological control purposes [24]. In the early 1990s, this species was introduced to Taiwan for scientific purposes. A million individuals were released for the biological control of spider mites at four sites—tea and fruit farms in central and southern Taiwan (e.g., strawberry fields in Miaoli County) [32,33,34]. Despite the clear evidence that *P. persilimis* is an efficient biological control agent, no individuals have been observed in comprehensive surveys of phytoseiids for over 30 years since the first mass release [21]. The failure to establish this species is worthy of investigation, especially in regard to future applications of biological control.

By assessing the suitability of areas (as habitats) in which *P. persimilis* was released in Taiwan in the 1990s, we sought to identify a mechanism that explains the failed establishment of this species. As *P. persimilis* originates from the Mediterranean basin [23,25,26], we assumed that temperature and precipitation were limiting factors, based on previous biological studies of *P. persimilis* [for example, 27–29]. Therefore, MaxEnt was used to predict the potential distribution of *P. persimilis* in Taiwan, with inferences made for habitat suitability under certain climatic conditions, including temperature and precipitation. We hypothesized that low habitat suitability would result in population collapse or failed establishment. The assessment framework can serve as a reference for the introduction of exotic natural enemies and their establishment and persistence. It can also help in the assessment of risks for the environment.

## 2. Materials and Methods

### 2.1. Species Occurrence Dataset

Global occurrence data on *P. persimilis* (Figure 1, Table S1) were collected from the Phytoseiidae Database [26], Phytoseiid Mite Portal [30], and a Global Biodiversity Information Facility dataset on the predator–prey system involving *P. persimilis* and *T. urticae* [24], including native and established population of *P. persilimis*. Next, we used Google Earth Pro to obtain the latitude–longitude coordinates of *P. persimilis* localities. Repeatedly acquiring data on occurrence may cause sampling bias, leading to increased density of the species distribution data in a certain area and resulting in deviations in the prediction results [35,36]. Therefore, we excluded duplicate and unclear distribution localities using Quantum GIS (QGIS) [37] and ensured Figure 1, the presence of only one distribution point in each raster to avoid overfitting. In total, 99 localities (Table S1) were included for analysis. The species is primarily distributed in countries in the Mediterranean basin, south and west Australia, western South America, and the western United States [24,25,26]. All distribution data were organized using Microsoft Excel, and in accordance with MaxEnt software format requirements were saved in a comma-separated values (CSV) file for further analysis [38].

### 2.2. Environmental Variables

We used a set of 19 environmental variables (current period: 1970–2000) from WorldClim (https://www.worldclim.org, accessed on 3 January 2021) [39] at a spatial resolution of 30 arc-seconds (1 km^2^). These variables were derived from monthly temperature and rainfall values and represented annual trends, seasonality, and extreme or restrictive environmental factors (Table S2). Bioclimatic variables are related to the distribution and survival of small arthropods and have been widely used in global studies concerning the prediction of species distribution [14,40]. We extracted data from the WorldClim database using RStudio (version 2.1) with R language ‘raster’ [41] and ‘rgdal’ [42] packages. 

### 2.3. Distribution Modeling

We used MaxEnt (v.3.4.1) [43] to predict the habitat suitability of *P. persimilis* in Taiwan based on global distribution data (Figure 1, Table S1) and environmental variables (Table 1) using the R package ‘dismo’ [44] in RStudio. We generated final models and the global potential distribution (Figure S1) of this species. In addition, the four release sites (Table S3) were mapped, to investigate the failed establishment of this species. 

To avoid or remove multicollinearity, environmental variables and species distribution data were imported into MaxEnt for the initial model to calculate the contribution rate using the jackknife test. Jackknifing was performed to determine the contribution of various bioclimatic variables to pest distribution prediction. Based on their contributions, we removed variables with relatively little importance, and the selected variables for further modeling included the minimum temperature of the coldest month (bio06), the mean diurnal range (bio02), precipitation of the warmest quarter (bio18), precipitation of the coldest quarter (bio19), the maximum temperature of the warmest month (bio5), the mean temperature of the wettest quarter (bio08), and the annual mean temperature (bio01) (Table 1). 

To avoid or reduce overfitting, the performances of diverse models with different sets of parameters (i.e., the feature type and regularization multiplier) were evaluated to determine the best fitting model using the R package ‘ENMeval’ [45]. In each model, we performed cross-variation in ten-fold replicates; based on the best model, other parameters were set as the default; among the five feature types (i.e., linear, quadratic, product, threshold, and hinge), linear and quadratic types were allowed, and the regularization multiplier was set to 0.5. We used presence-only data to generate pseudo-absences, and 10,000 background points were randomly selected by the MaxEnt model, which either ran 500 iterations of these processes or continued until a convergence threshold of 0.00001 was attained.

The prediction results from MaxEnt modelling were evaluated according to threshold-independent area under curve (AUC) values calculated using the R program. Receiver operating characteristic (ROC) curves were used to plot the true-positive rate against the false-positive rate, and AUC was used as a measure of the goodness of fit of the model [10,46]. We selected a test sensitivity of 0% and 10% omission rates (OR) [47,48]. The AUC value ranges from 0 to 1, with higher values indicating higher predictive performance [49]. In the case of default OR, the value at 10% was 0.10, and the sensitivity test value at 0% was 0; poor performance was indicated by a value exceeding the predicted rate [50]. The logistic output was chosen as an estimate of the probability of the presence conditioned by the environmental variables (i.e., habitat suitability), per grid cell. Jackknifing was used to screen for dominant environmental variables [51]. In addition, we obtained response curves showing single effects of individual variables on the species occurrence and the curves for all environmental variables (i.e., selected based on the initial modelling).

## 3. Results

### 3.1. Comparison of Suitable Distributions of P. persimilis and Four Release Sites in Taiwan

**Figure 2:**
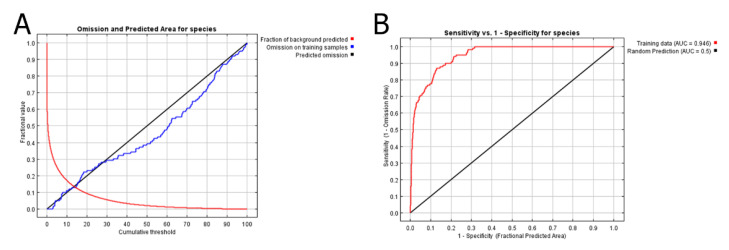
Predictions of *Phytoseiulus* *persimilis*: (**A**) omission rate, (**B**) ROC curve and AUC value.

**Figure 3:**
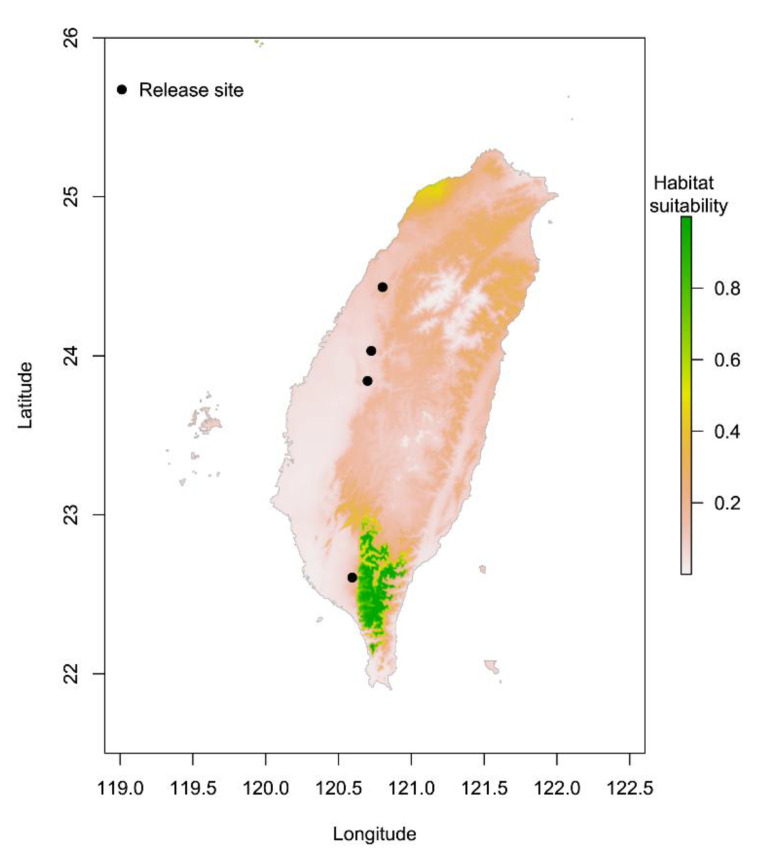
Comparison of potential distribution areas and four release sites of *Phytoseiulus persimilis* in Taiwan.

The OR of *P.* *persimilis* distribution prediction is shown in Figure 2. The training set OR (blue line); that is the cumulative threshold definition, should be close to the predicted OR (black line). The average OR of the training set for *P. persimilis* was 0.000. The ROC curve output from MaxEnt showed that the AUC of the *P. persimilis* training set was 0.946. According to the standards by which the AUC was evaluated, the prediction model showed high performance [50,52]. The potential distribution areas are shown in Figure 3. Green represents high habitat suitability, red represents moderate habitat suitability, and white represents low habitat suitability. The prediction revealed areas that would be highly suited with regard to climatic characteristics, namely the south-central mountainous areas of Kaohsiung City, Pingtung County, and Taitung County. The rest of the land area of Taiwan was unsuitable habitat for *P. persimilis*. The four release sites, located in low elevation areas in central and southwestern Taiwan, were as follows: a strawberry farm in Dahu Township, Miaoli County [32]; a tea farm in Songbokeng, Mingjiang Township, Nantou County [33]; a papaya farm in Wufeng District, Taichung City [34], and a papaya farm in Daliao District, Kaohsiung City [34] (Figure 3). These locations showed low occurrence probabilities of 0.11, 0.09, 0.05, and 0.02, respectively (Table S3), indicating that these areas were poorly suited to the establishment of *P. persimilis* populations. In addition, the potential distributions of *P. persimilis* in Southeast Asia, and worldwide are shown in Figure S1 and Figure S2.

### 3.2. Environmental Variables Influencing the Distribution of Phytoseiulus persimilis

**Figure 4:**
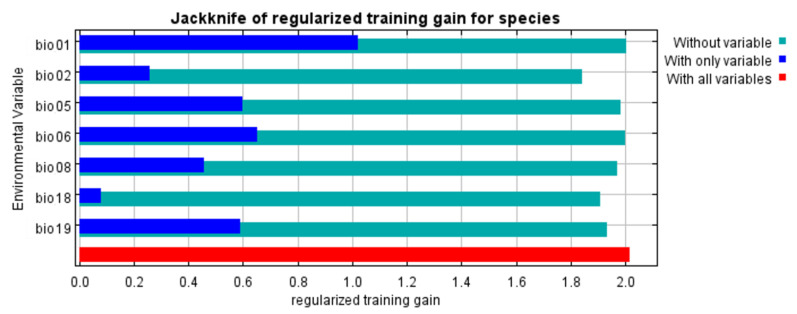
Relative importance of seven selected environmental variables of *Phytoseiulus persimilis* based on the jackknife test.

The contribution rate of environmental variables and their permutation importance according to the jackknife test are presented in Figure 4 and Table 1. The minimum temperature of the coldest month (bio06), the mean diurnal range (bio02), and precipitation of the warmest quarter (bio18), all exceeded 10% (44.7%, 24.8%, and 10.6%, respectively). Moreover, these variables accounted for 80.1% of the cumulative contribution rate, which was the highest proportion among the variables. The permutation importance of bio06 (25.2%) was the highest, followed by that of the annual mean temperature (bio01), the maximum temperature of the warmest month (bio05), and the mean temperature of the wettest quarter (bio08) (21.8%, 19.5%, 18.8%, respectively). Overall, the permutation importance of these four variables was over 10%, which is strongly indicative of the modeling results.

**Figure 5:**
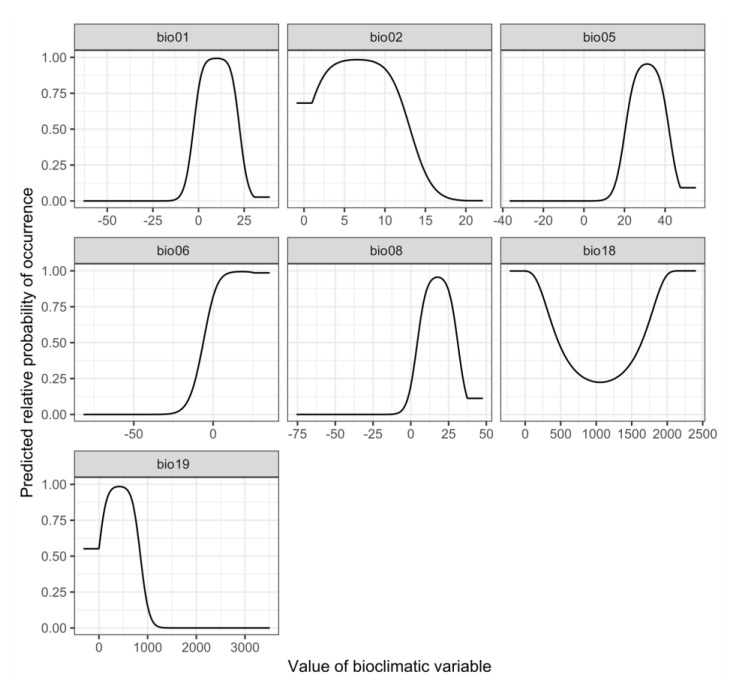
Response curves for seven selected environmental variables of *Phytoseiulus persimilis.*

Response curves for seven selected environmental variables of *Phytoseiulus persimilis* are shown in Figure 5, showing several relationship patterns (e.g., unimodal and monotonically increasing or decreasing). For instance, the minimum temperature of the coldest month (bio06) was the most contributing variable and exhibited a monotonically increasing pattern against the occurrence of *P. persimilis*. The mean diurnal range (bio02) showed a unimodal pattern against the occurrence of *P. persimilis*. In addition, the probability of occurrence showed a U-shaped pattern with respect to precipitation of the warmest quarter (bio18).

## 4. Discussion

MaxEnt has been widely applied in invasive pest studies, by assessing global occurrence data to predict local potential distribution before invasion, e.g., [15,16]. To the best of our knowledge, the present study is the first to use MaxEnt to predict habitat suitability for the establishment of exotic natural enemies and the first such study on phytoseiid mites. Our hypothesis is that temperature and precipitation are limiting factors based on previous biological studies of establishment failure of *P. persimilis, e.g.*, [27,28,29]. We input current global distribution data of *Phytoseiulus persimilis* Athias-Henriot into MaxEnt for potential distributions in Taiwan and compared the data with previous release sites to determine the reasons for the failure of the establishment of this species after its introduction in the 1990s. The prediction results were favorable, as indicated by the average OR (0.000) and AUC of the training set (0.946). These results are promising for the practical application of MaxEnt in the introduction of exotic natural enemies. Our study showed a novel concept of modeling validation when establishment failure occurred at sites with low predicted values of habitat suitability. This provides a plausible mechanism for the failure based on environmental factors. The goals of our study were to elucidate this mechanism and provide information for the practical application of biocontrol agents. 

Although the study model demonstrated high accuracy, numerous uncertainties can affect the prediction results, including sample size and bias (species distribution sites), background data (pseudo-absence), operational methods, explanatory variables, and study scale [45,53]. For example, the prediction accuracy increases with an increase in sample size; however, this occurs only up to a point; it decreases when an overly large number of samples are used [45,54]. In addition, the number of released populations, releasing methods, and natural enemies (e.g., microbes) of biological control agents may also affect establishment failure. Future studies should examine the reasons for the establishment of exotic biological control agents based on all cases of classical biological control, and evaluation of the results of such studies will help reduce the risk posed by biological control agents to the environment.

### 4.1. Potential Suitable Areas for P. persimilis and the Four Release Sites

*P. persimilis* was introduced into Taiwan in the 1990s, with a million individuals released to control spider mites at four localities in central and southwestern Taiwan [32,33,34]. However, no individuals have been observed since then. We assumed that temperature and precipitation limit the establishment of this species. Therefore, MaxEnt was used to predict the potentially suitable distribution of *P. persimilis* in Taiwan. In Figure 3, we present a comparison of this distribution with the four release sites. Suitable areas are distributed throughout the mountainous areas of southern Taiwan. However, three of the release localities were in central Taiwan, and one locality was in southwestern Taiwan. The results suggest that establishment failure can be ascribed to the fact that previous release sites were unsuitable for *P. persimilis* (habitat suitability of 0.11, 0.09, 0.05, and 0.02, respectively). The climate in the suitable areas is similar to the Mediterranean climate; in contrast, southwestern Taiwan has a tropical climate, with higher temperatures and lower precipitation in winter. Therefore, the prediction results supported our hypothesis; the main reason is that previous release localities are low-suitability areas.

*P. persimilis* has been commercialized since 1995 in Japan and is widely released in the field and in greenhouses for controlling spider mites [44]. Based on the survey results of Toyoshima et al. [30], predatory mites have been widely distributed in Japan, including in Chiba, Kanagawa, Shizuoka, Kagoshima, and Okinawa. As illustrated in Figure S1, the prediction showed that Japan is suitable for the species, which is consistent with the establishment conditions based on the investigation.

With regard to the establishment of exotic natural enemies, considerations beyond climate should be taken into account, including the release frequency, release number, diet and food sources, as well as intraguild interactions among natural enemy species [1,2,4]. First, *P. persimilis* is a specialized predator of *Tetranychus* mites; thus, the population persistence of this predator is dependent on the availability of a sufficient amount of prey [20,25,55]. Moreover, in a study conducted in Spanish clementine orchards, Abad-Moyano et al. [56] reported that *Euseius stipulatus* (Athias-Henriot) adversely affected the establishment of *N. californicus* and *P. persimilis*, which in turn hindered the control of *T. urticae*. In addition, the cultivation method may be another element that affects the establishment of exotic natural enemies. As mentioned previously, *P. persimilis* was released at a strawberry farm. Fallowing and crop rotation affect spider mite populations, thereby adversely affecting the persistence of its natural enemies.

### 4.2. Environmental Variables Affect the Establishment of P. pesimilis

*P. persimilis* originates from Algeria [23] and is primarily distributed in the Mediterranean basin [24,25,26], where the climate is characterized by mild rainy winters and warm to hot dry summers [27]. Previous studies have revealed that temperature and precipitation directly affect the growth and distribution of *P. persimilis*, which thrives in warm and highly humid environments [27,28,29]. This is consistent with our prediction results, and the potential distribution closely matched that in areas with Mediterranean climates worldwide (Figure S1), whereas almost all regions of Southeast Asia (Figure S2) would be unsuitable for *P. persimilis*.

This study showed that key climatic factors affecting the distribution of *P. persimilis*, namely bio06, bio02, bio18, and bio19, were selected based on their contribution rates, with their contribution of 80.1% (Table 1). The minimum temperature of the coldest month (bio06) and the mean diurnal range (bio02) had the most significant effects on the distribution. The response curve of bio06 revealed that the coldest temperature was the limiting factor for the distribution of *P. persimilis*. A monotonically increasing pattern was observed after 0 °C was 1. We considered that this could be related to the cold hardiness of *P. persimilis* (no diapause) [29,57,58]. The mean diurnal range (bio02) presented a unimodal pattern, which indicated that habitat suitability was 1 when the temperature range between 5 and 10 °C. Skirvin and Fenlon [29] reported that *P. persimilis* consumed more prey at temperatures between 15 and 25 °C but consumed less when the temperature was 30 °C. The next two variables were precipitation of the coldest and warmest quarter (bio18 and bio19). The response curve of bio18 showed that the occurrence probability was low when the precipitation was between 500 and 1500 mm. In contrast, the response curve of bio19 showed that the occurrence probability was high in the precipitation between 200 and 800 mm, but dramatically decreased when precipitation reached 1000 mm. Stenseth [27] reported that the relative humidity required for sustaining *P. persimilis* populations ranges from 40% to 80%, and this result is consistent with the unimodal patterns of bio19. Although the species prefers a climate with high humidity, very high humidity negatively affects their survival. 

### 4.3. Necessity of Predicting the Potential Distributions of Exotic Natural Enemies

Many natural enemies have provided successful results in biological control programs around the world, including tropical areas [5,6]. Classical biological control involves the introduction and establishment of exotic natural enemies for permanent pest control, whereas augmentative biological control involves the supplemental release of exotic natural enemies; for example, through inoculation and inundation, when the current number is insufficient for effective pest control [1,2,3,4]. Exotic natural enemies are typically introduced for pest control; therefore, the determination of factors affecting their establishment and the risks entailed in their use is essential [1,2,4,9]. Regardless of whether successful establishment is desired, the prediction of potentially suitable release sites must be included in the risk assessment. If a biological control program requires the persistence of exotic natural enemies, they must be released in suitable areas. 

## 5. Conclusions

The establishment of exotic natural enemies released into the field for pest control warrants urgent attention, as does the assessment of risks they pose to endemic populations and the possibility of establishment [1,2,4,9]. The prediction of potentially suitable release sites must be conducted to increase the possibility of establishment. The present findings serve as a basis for optimizing the population persistence of exotic natural enemies. Alternatively, the failure to establish a population provides an opportunity to reveal a possible strategy to determine eco-safety when exotic natural enemies are introduced in a particular area as biological control agents for pests but are unable to maintain their populations in the regions where they are released. 

Our study is the first to use the machine learning method MaxEnt to predict suitable distribution of exotic natural enemies and is the first such study on predatory mites based on environmental variables. According to the present results, suitable areas of *P. persimilis* are located in northern Taiwan as well as in the mountainous areas of eastern Taiwan. The reason for the failure of the previous four releases could be that the release took place in unsuitable areas (if occurrence is primarily or only determined by these environmental factors). Therefore, if the purpose of releasing biological control agents is to establish a population that will persist in the field, the control agents need to be released in northern and eastern Taiwan. In addition, temperature and precipitation played crucial roles in the predicted distribution. This result is consistent with previous biological studies of *P. persimilis*, e.g., [27,28,29], which show that the species thrives in a Mediterranean climate. We believe that the practical application of machine learning in determining mite distribution is the first step in the risk assessment of biological control agents. In future studies, the simulation of all previous cases of introductions of exotic biological control agents should be conducted to elucidate the factors for establishment, and the findings of such studies will help reduce the risk of damage by biological control agents to the environment. In addition, other ecological factors, such as species interactions, should be part of the risk assessment in the real, complex world.

## Figures and Tables

**Table 1:** Contribution rate and permutation importance of selected environment variables.

Variable Codes	Environment Variables	Contribution Rate %	Permutation Importance %
bio06	Min temperature of coldest month (°C)	44.7	25.2
bio02	Mean diurnal range (°C)	24.8	8.1
bio18	Precipitation of warmest quarter (mm)	10.6	5
bio19	Precipitation of coldest quarter (mm)	6.4	1.7
bio05	Max temperature of warmest month (°C)	5.7	19.5
bio08	Mean temperature of wettest quarter (°C)	4.4	18.8
bio01	Annual mean temperature (°C)	3.5	21.9

## Data Availability

Please see the section “Supplementary Materials”.

## Supplementary Materials

The following are available online at https://www.mdpi.com/article/10.3390/insects12050418/s1, Figure S1: potential distribution areas of Phytoseiulus persimilis in the world, Figure S2: potential distribution areas of Phytoseiulus persimilis in southeast Asia, Table S1: occurrence data of Phytoseiulus persimilis, Table S2: contribution rate and permutation importance of environment variables, Table S3: release sites of Phytoseiulus persimilis in Taiwan.

## References

[1] van Lenteren J., Babendreier D., Bigler F., Burgio G., Hokkanen H.M.T., Kuske S., Loomans A.J.M., Menzler-Hokkanen I., van Rijn P.C.J., Thomas M.B. (2003). Environmental risk assessment of exotic natural enemies used in inundative biological control. BioControl.

[2] van Lenteren J.C., Bale J., Bigler F., Hokkanen H.M.T., Loomans A.J.M. (2006). Assessing risks of releasing exotic biological control agents of arthropod pests. Annu. Rev. Entomol..

[3] Cock M.J.W., van Lenteren J.C., Brodeur J., Barrat B.I.P., Bigler F., Bolckmans K., Cônsoli F.L., Haas F., Mason P.G., Parra J.R.P. (2010). Do new access and benefit sharing procedures under the convention on biological diversity threaten the future of biological control?. BioControl.

[4] Hajek A.E., Hurley B.P., Kenis M., Garnas J.R., Bush S.J., Wingfield M.J., van Lenteren J.C., Cock M.J.W. (2016). Exotic biological control agents: A solution or contribution to arthropod invasions?. Biol. Invasions.

[5] Wyckhuys K.A.G., Hughes A.C., Buamas C., Johnson A.C., Vasseur L., Reymondin L., Deguine J.-P., Sheil D. (2019). Biological control of an agricultural pest protects tropical forests. Commun. Biol..

[6] Kaufman L.V., Yalemar J., Wright W.G. (2020). Classical biological control of the erythrina gall wasp, *Quadrastichus erythrinae*, in Hawaii: Conserving an endangered habitat. Biol. Control.

[7] Messelink G.J., Bennison J., Alomar O., Ingegno B.L., Tavella L., Shipp L., Palevsky E., Wäckers F.L. (2014). Approaches to conserving natural enemy populations in greenhouse crops: Current methods and future prospects. BioControl.

[8] van Lenteren J.C., Loomans A.J.M., Babendreier D., Bigler F., Roy H.E., Wajnberg E. (2007). Harmonia axyridis: An environmental risk assessment for Northwest Europe. From Biological Control to Invasion: The Ladybird Harmonia Axyridis as a Model Species.

[9] Loomans A.J.M. (2021). Every generalist biological control agent requires a special risk assessment. BioControl.

[10] Phillips S.J., Anderson R.P., Schapire R.E. (2006). Maximum entropy modeling of species geographic distributions. Ecol. Model..

[11] Elith J., Phillips S.J., Hastie T., Dudík M., Chee Y.E., Yates C.J. (2011). A statistical explanation of MaxEnt for ecologists. Divers. Distrib..

[12] Qiao H.J., Hu J.H., Huang J.H. (2013). Theoretical basis, future directions, and challenges for ecological niche models. Sci. Sin. Vitae.

[13] Barbet-Massin M., Rome Q., Villemant C., Courchamp F. (2018). Can species distribution models really predict the expansion of invasive species?. PLoS ONE.

[14] Xu D., Li X., Jin Y., Zhuo Z., Yang H., Hu J., Wang R. (2020). Influence of climatic factors on the potential distribution of pest *Heortia vitessoides* Moore in China. Glob. Ecol. Conserv..

[15] Jarnevich C.S., Young N., Venette R.C. (2015). Using the MAXENT program for species distribution modelling to assess invasion risk. Pest Risk Modelling and Mapping for Invasive Alien Species.

[16] Tang J., Li J., Lu H., Lu F., Lu B. (2019). Potential distribution of an invasive pest, *Euplatypus parallelus*, in China as predicted by Maxent. Pest. Manag. Sci..

[17] Slatculescu A.M., Clow K.M., McKay R., Talbot B., Logan J.J., Thickstun C.R., Jardine C.M., Ogden N.H., Knudby A.J., Kulkarni M.A. (2020). Species distribution models for the eastern blacklegged tick, *Ixodes scapularis*, and the Lyme disease pathogen, *Borrelia burgdorferi*, in Ontario, Canada. PLoS ONE.

[18] Negrini M., Fidelis E.G., Picanço M.C., Ramos R.S. (2020). Mapping of the *Steneotarsonemus spinki* invasion risk in suitable areas for rice (*Oryza sativa*) cultivation using MaxEnt. Exp. Appl. Acarol..

[19] Chant D.A., McMurtry J.A. (2007). Illustrated Keys and Diagnoses for the Genera and Subgenera of the Phytoseiidae of the World (Acari: Mesostigmata).

[20] McMurtry J.A., De Moraes G.J., Sourassou N.F. (2013). Revision of the lifestyles of phytoseiid mites (Acari: Phytoseiidae) and implications for biological control strategies. Syst. Appl. Acarol..

[21] Liao J.R., Ho C.C., Lee H.C., Ko C.C. (2020). Phytoseiidae of Taiwan (Acari: Mesostigmata).

[22] EPPO List of Biological Control Agents Widely Used in the EPPO Region: PM6/3(2) 2020 Version. https://www.eppo.int/RESOURCES/eppo_standards/pm6_biocontrol.

[23] Athias-Henriot C. (1957). Phytoseiidae et Aceosejidae (Acarina, Gamasina) d’Algerie. I. Genres *Blattisocius* Keegan, *Iphiseius* Berlese, *Amblyseius* Berlese, *Phytoseius* Ribaga, *Phytoseiulus* Evans. Bull. Soc. Hist. Nat. Afr. N.L..

[24] Migeon A., Tixier M.-S., Navajas M., Litskas V.D., Stavrinides M.C. (2019). A predator-prey system: *Phytoseiulus persimilis* (Acari: Phytoseiidae) and *Tetranychus urticae* (Acari: Tetranychidae): Worldwide occurrence datasets. Acarologia.

[25] Takahashi F., Chant D.A. (1993). Phylogenetic relationships in the genus *Phytoseiulus* Evans (Acari: Phytoseiidae). II. Taxonomic review. Int. J. Acarol..

[26] Demite P.R., De Moraes G.J., McMurtry J.A., Denmark H.A., Castilho R.C. Phytoseiidae Database. http://www.lea.esalq.usp.br/phytoseiidae/.

[27] Stenseth C. (1979). Effect of temperature and humidity on the development of Phytoseiulus Persimilis and its ability to regulate populations of Tetranychus urticae [Acarina: Phytoseiidae. Tetranychidae]. Entomophaga.

[28] Laing J.E. (1968). Life history and life table of *Phytoseiulus persimilis*. Acarologia.

[29] Skirvin D.J., Fenlon J.S. (2003). The effect of temperature on the functional response of *Phytoseiulus persimilis* (Acari: Phytoseiidae). Exp. Appl. Acarol..

[30] Toyoshima S., Kishimoto H., Amano H. Phytoseiid Mite Portal. https://phytoseiidae.acarology-japan.org/.

[31] Rasmy A.H., Ellaithy A.Y.M. (1988). Introduction of *Phytoseiulus persimilis* for two spotted spider mite control in greenhouses in Egypt [Acari: Phytoseiidae, tetranychidae]. Entomophaga.

[32] Lee W.T., Lo K.C. (1989). Integrated control of two-spotted spider mite on strawberry in Taiwan. Chinese J. Entomol. Special Publ..

[33] Ho C.C. (1990). A preliminary study on the biological control of *Tetranychus kanzawai* in tea field by *Amblyseius fallacis* and *Phytoseiulus persimilis* (Acarina: Tetranychidae, Phytoseiidae). J. Agric. Res. China.

[34] Hao H.H., Wang H.L., Lee W.T., Lo K.C. (1996). Studies on biological control of spider mites on papaya. J. Agric. Res. China.

[35] Peterson A.T., Papes M., Eaton M. (2007). Transferability and model evaluation in ecological niche modeling: A comparison of GARP and Maxent. Ecography.

[36] Fourcade Y., Engler J.O., Rödder D., Secondi J. (2014). Mapping species distributions with MAXENT using a geographically biased sample of presence data: A performance assessment of methods for correcting sampling bias. PLoS ONE.

[37] QGIS Development Team QGIS Geographic Information System. Open Source Geospatial Foundation Project..

[38] Phillips S.J. A Brief Tutorial on MAXENT. http://biodiversityinformatics.amnh.org/open_source/maxent/.

[39] Fick S.E., Hijmans R.J. (2017). WorldClim 2: New 1 km spatial resolution climate surfaces for global land areas. Int. J. Climatol..

[40] De Meyer M., Robertson M.P., Mansell M.W., Ekesi S., Tsuruta K., Mwaiko W., Vayssières J.-F., Peterson A.T. (2010). Ecological niche and potential geographic distribution of the invasive fruit fly *Bactrocera invadens* (Diptera, Tephritidae). Bull. Entomol. Res..

[41] Hijmans R. Package “Raster”. http://raster.r-forge.r-project.org/.

[42] Bivand R., Keitt T., Rowlingson B. Package “Rgdal”. https://CRAN.R-project.org/package=rgdal.

[43] Phillips S.J., Dudik M., Schapire R.E. Maxent Software for Modeling Species Niches and Distribution (Version 3.4.1). https://biodiversityinformatics.amnh.org/open_source/maxent/.

[44] Hijmans R.J., Phillips S., Leathwick J., Elith J. Package ‘Dismo’. http://cran.r-project.org/web/packages/dismo/index.html.

[45] Muscarella R., Galante P.J., Soley-Guardia M., Boria R.A., Kass J.M., Uriarte M., Anderson R.P. (2014). ENMeval: An R package for conducting spatially independent evaluations and estimating optimal model complexity for Maxent ecological niche models. Methods Ecol. Evol..

[46] Hernandez P.A., Graham C.H., Master L.L., Albert D.L. (2006). The effect of sample size and species characteristics on performance of different species distribution modelling methods. Ecography.

[47] Peterson A.T., Papeş M., Soberón J. (2008). Rethinking receiver operating characteristic analysis applications in ecological niche modeling. Ecol. Model..

[48] Liu C., White M., Newell G. (2013). Selecting thresholds for the prediction of species occurrence with presence-only data. J. Biogeogr..

[49] Kumar S., Neven L.G., Zhu H., Zhang R. (2015). Assessing the global risk of establishment of *Cydia pomonella* (Lepidoptera: Tortricidae) using CLIMEX and MaxEnt niche models. J. Econ. Entomol..

[50] Peterson A.T., Soberón J., Anderson R.P., Pearson R.G., Martínez-Meyer E., Nakamura M., Araújo M.B. (2011). Ecological Niches and Geographic Distributions: A Modeling Perspective.

[51] Boria R.A., Olson L.E., Goodman S.M., Anderson R.P. (2014). Spatial filtering to reduce sampling bias can improve the performance of ecological niche models. Ecol. Model..

[52] Velasco J.A., Gonzalez-Salazar C. (2019). Akaike information criterion should not be a “test” of geographical prediction accuracy in ecological niche modelling. Ecol. Inform..

[53] Jayasinghe S.L., Kumar L. (2019). Modeling the climate suitability of tea [*Camellia sinensis* (L.) O. Kuntze] in Sri Lanka in response to current and future climate change scenarios. Agric. For. Meteorol..

[54] Feeley K.J., Silman M.R. (2011). Keep collecting: Accurate species distribution modelling requires more collections than previously thought. Divers. Distrib..

[55] Elith J., Graham C.H., Anderson R.P., Dudík M., Ferrier S., Guisan A., Hijmans R.J., Huettmann F., Leathwick J.R., Lehmann A. (2006). Novel methods improve prediction of species’ distributions from occurrence data. Ecography.

[56] Kondo A. (2004). Colonizing characteristics of two phytoseiid mites, *Phytoseiulus persimilis* Athias-Henriot and *Neoseiulus womersleyi* (Schicha) (Acari: Phytoseiidae) on greenhouse grapevine and effects of their release on the kanzawa spider mite, *Tetranychus kanzawai* Kishida (Acari: Tetranychidae). Appl. Entomol. Zool..

[57] Abad-Moyano R., Urbaneja A., Schausberger P. (2010). Intraguild interactions between Euseius stipulatus and the candidate biocontrol agents of Tetranychus urticae in Spanish clementine orchards: *Phytoseiulus persimilis* and *Neoseiulus californicus*. Exp. Appl. Acarol..

[58] Morewood W.D. (1993). Diapause and cold hardiness of phytoseiid mites (Acarina: Phytoseiidae). Eur. J. Entomol..

