# Supplementary figures and images for: Niche Modeling May Explain the Historical Population Failure of Phytoseiulus persimilis in Taiwan: Implications of Biocontrol Strategies
Source: Insects. 2021 May 6;12(5):418. doi: 10.3390/insects12050418 (PMC8148512; doi:10.3390/insects12050418)

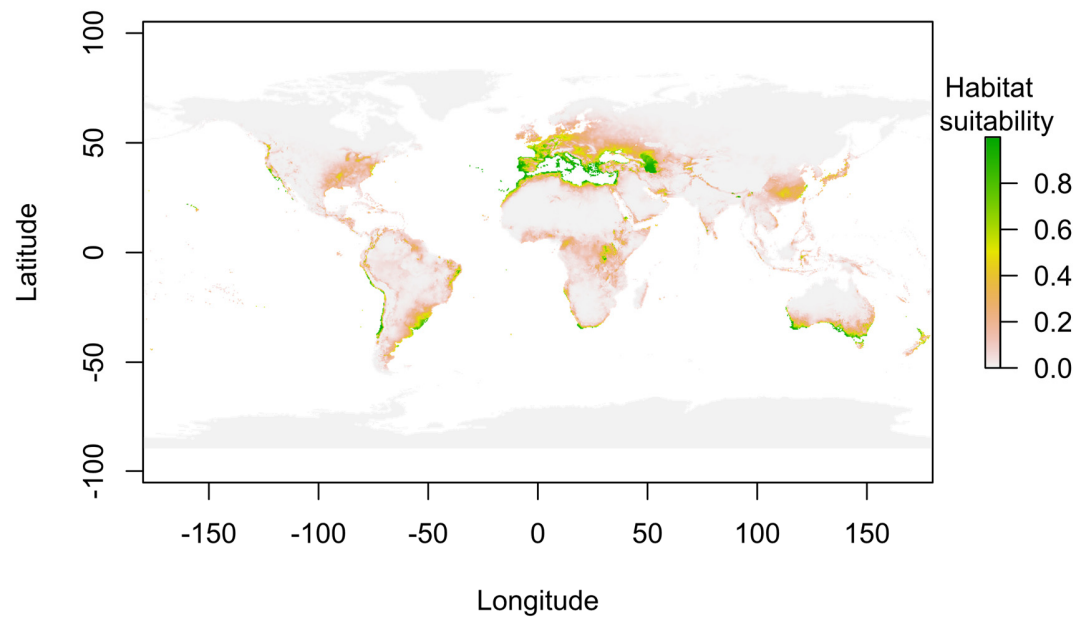

**Figure S1.** Potential distribution areas of *Phytoseiulus persimilis* in the world.

Supplement: Supplementary file 1 [file insects-12-00418-s001.zip › Figure_S1.pdf]

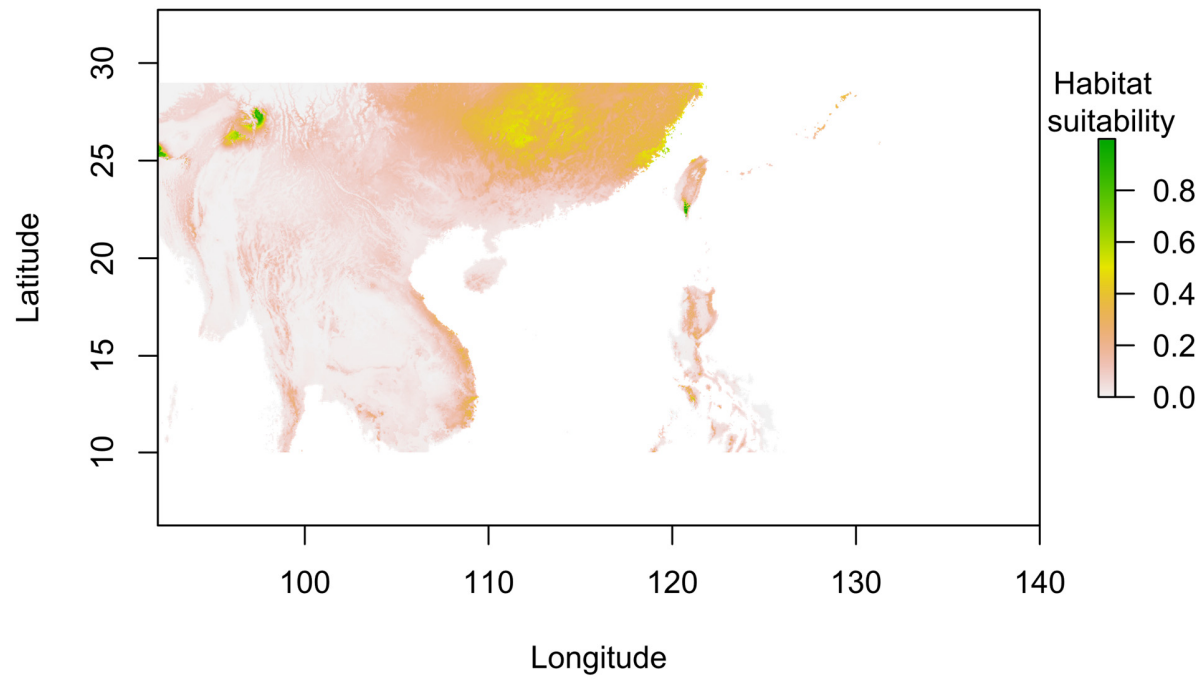

**Figure S2.** Potential distribution areas of *Phytoseiulus persimilis* in southeast Asia.

Supplement: Supplementary file 1 [file insects-12-00418-s001.zip › Figure_S2.pdf]
